# Special Issue: Nanobody

**DOI:** 10.3390/antib9010006

**Published:** 2020-03-06

**Authors:** Patrick Chames, Ulrich Rothbauer

**Affiliations:** 1 Aix Marseille University, CNRS, INSERM, Institute Paoli-Calmettes, CRCM, 13009 Marseille, France; 2 Pharmaceutical Biotechnology, Eberhard Karls University, 72076 Tuebingen, Germany; 3 Natural and Medical Sciences Institute at the University of Tuebingen, Markwiesenstr. 55, 72770 Reutlingen, Germany

Since their first description in 1993 [1], single-domain antibody fragments derived from heavy-chain-only antibodies of camelids have received increasing attention as highly versatile binding molecules in the fields of biotechnology and medicine. The term “nanobody”—originally introduced as a trademark of the company Ablynx in 2003—became the general label for those proteins, perfectly reflecting their small dimensional size (2.5 nm × 4 nm; ~13 kDa). Since the expiration of main patent claims in 2013, there has been an emerging tendency in commercializing nanobodies as research, diagnostic and therapy agents.

Nanobodies can be efficiently selected from large (semi-) synthetic/naive or immunized cDNA-libraries using well established display technologies like phage- or yeast-display [2,3]. The simple and single-gene format enables the production of purified nanobodies in the mg–g range per liter of culture, thereby offering an unlimited supply of consistent binding molecules. Additionally, nanobodies can be easily genetically or chemically engineered. Nanobodies are characterized by high affinities and specificities, robust structures, including stable and soluble behaviors in hydrophilic environments and superior cryptic cleft accessibility, low-off target accumulation, and deep tissue penetration [4]. To date, many nanobodies have been evolved into versatile research and diagnostic tools and the list of therapeutic nanobodies applied in clinical trials is constantly growing [5]. Nanobody-derived formats comprise the nanobody itself, homo- or heteromultimers, nanobody-coated nanoparticles or matrixes, nanobody-displayed bacteriophages or enzymatic-, fluorescent- or radionuclide-labeled nanobodies. All these formats were successfully applied in basic biomedical research, cellular and molecular imaging, diagnosis or targeted drug delivery and therapy. With caplacizumab from Sanofi, the first therapeutic active nanobody, was approved by the FDA in February 2019 [6].

This Special Issue on “Nanobodies” includes original manuscripts and reviews covering various aspects related to the discovery, characterization, engineering and application of nanobodies for biomedical research, diagnostics and therapy.

Starting a series of original articles, Longhin et al. selected a set of six novel nanobodies from an immunized library directed against the zinc-transporting P_IB_-ATPase ZntA from *Shigella sonnei* (SsZntA). Further exploiting their ability of bind to cavities and active sites of the target protein, with Nb9, the authors identified a highly selective inhibitor of the ATPase activity of SsZntA. These nanobodies provide a versatile toolset for structural and functional studies of this subset of ATPases [7]. Focusing on more therapeutic application, nanobodies can be a rich source of neutralizing anti-viral reagents. Liu et al. selected a panel of high affinity nanobodies against the E2/E3E2 envelope protein of the Western equine encephalitis virus (WEEV) and demonstrated their potential as detection reagents. The intrinsic modularity and stability of such nanobodies might also be exploited to create stable neutralizing molecules adapted to storage in resource-limited areas [8]. Similarly, Ramage et al. used alpacas immunized with recombinant hemagglutinin from two representative Influenza B viruses to generate nanobodies with both cross-reactive and lineage-specific binding, and carefully analyzed their specificities over a large panel of viruses. The broadly reactive nanobodies might have interesting applications in Influenza B virus diagnostics, vaccine potency testing and possibly as neutralizing immunotherapeutics with potential for intranasal delivery [9]. Exploiting a similar concept, Strokappe et al. generated a panel of neutralizing nanobodies targeting the HIV gp41 and gp120 envelope proteins, thereby describing three new epitopes on these targets. Interestingly, using detailed biophysical and structural characterization, the author took advantage of the modularity of nanobodies to successfully design bispecific constructs with up to 1400-fold higher neutralization potencies than the mixture of the individual nanobodies, thus endowed with a high therapeutic or microbicide potential [10]. Nanobodies also have therapeutic potential beyond virology. In this issue, Heukers et al. took advantage of the small size of nanobodies to generate a new generation of biopharmaceuticals with nanomolar potency by combining anti-hepatocyte growth factor receptor nanobodies to a photosensitizer, thus allowing efficient targeted photodynamic therapy upon local illumination [11]. A detailed epitope mapping is extremely helpful for downstream applications of nanobodies. In their study, Angalakurthi and colleagues used hydrogen exchange-mass spectrometry (HX-MS) to identify the epitopes of 21 nanobodies directed against the ribosome-inactivating subunit (RTA) of ricin toxin. Modelling these epitopes on the surface of RTA not only showed the potential of HX-MS to identify three dimensional epitopes but also supports the generation of a comprehensive B-cell epitope map of ricin toxin [12]. One of the most important features of nanobodies is that they can be genetically engineered for their desired downstream application. In this context, Anderson et al. demonstrated the potential of nanobodies fused to Beta-galactosidase to detect antigens in immunoassays. Using the example of a nanobody specific for the Bacillus collagen-like protein of anthracis (BclA), the authors highlight the potential to engineer nanobodies as highly sensitive reagents for one-step detection of antigen spores in sandwich immunoassays [13].

To generate an intracellular biosensor which monitors the activation of RHO-GTPases, Laura Keller et al. selected a nanobody (RH57) specifically for the GTP-bound version of RHO-GTPase from a synthetic library. When expressed as a fluorescent fusion protein (chromobody), it visualizes the localization of activated endogenous RHO at the plasma membrane without interfering with signaling. As a BRET-based biosensor, the RH57 nanobody was able to monitor RHO spatio-temporal resolved activation in living cells [14]. To optimize the expression of such chromobodies for antigen visualization in living cells, Bettina Keller and colleagues presented a strategy to stabilize biosensors introduced into various cell lines. By site-directed integration of antigen sensitive chromobodies into the AAVS1 safe harbor locus of human cells using CRISPR/Cas9 gene editing, they generated stable chromobody cell lines which not only visualize the localization of the endogenous antigen but can also be used to monitor changes in antigen concentration by quantitative imaging [15]. Nanobodies fused to fluorescent proteins can also be applied for preclinical in vivo imaging. In this context, Gorshkova et al. generated and produced two previously reported TNF-α specific nanobodies fused to the far-red fluorescent protein Katushka. They evaluated the ability of both fluorescently labeled nanobodies to bind and neutralize TNF-α in vitro and to serve as fluorescent probes for in vitro and non-invasive molecular in vivo imaging. In addition to the visualization of local expression of TNF-α, they demonstrated that in vivo fluorescence of the engineered nanobodies correlates with TNF levels in living mice [16].

This set of original work is further complemented by a series of reviews highlighting the emerging potential of nanobodies in biomedical research, diagnostics and therapy. Aguilar and colleagues, the pioneers in the field, summarized recent developments on how intracellularly functional nanobodies combined with functional or structural units can be used to study and manipulate protein function in multicellular organisms and developmental biology [17]. As exemplified by several studies in this Special Issue, nanobodies open new avenues for the treatment of viral infections. De Vlieger et al. presented here an overview of the literature covering the use of nanobodies and derived formats to combat viruses including influenza viruses, human immunodeficiency virus-1, and human respiratory syncytial virus [18]. Jank et al. described another field of applications of nanobodies, namely their use as diagnostic and therapeutic reagents against stroke. They covered the advantages of nanobodies over conventional antibody-based therapeutics in the context of brain ischemia and described several innovative nanobody-based treatment protocols aiming at improving stroke diagnostic and therapy [19]. Exploring another very promising and new therapeutic field afforded by the peculiar nature of nanobodies, Bélanger et al. presented the most recent advances in the development of nanobodies as potential therapeutics across brain barriers, including their use for the delivery of biologics across the blood–brain and blood–cerebrospinal fluid barriers, the treatment of neurodegenerative diseases and the molecular imaging of brain targets [20]. Highlighting the unique potential and increasing applications of nanobodies for in vivo imaging, Pieterjan Debie and colleagues presented a comprehensive overview on the current state of the art on how to generate, functionalize and apply nanobodies as molecular tracers for nuclear imaging and image-guided surgery [21]. Finally, Chanier and Chames provided an in-depth coverage of the use of nanobodies as innovative building blocks providing new solutions for the detection and imaging of cancer cells, as well as the development of next-generation cancer immunotherapy approaches, including multispecific constructs for effector cell retargeting, cytokine and immune checkpoint blockade, cargo delivery or the design of optimized CAR T cells [22]. 

We are convinced that this collection of articles will provide novel insights and information which are valuable to many readers working on different aspects of nanobodies. The editors would like to thank all the contributors for their excellent submissions to this Special Issue, as well as the reviewers and the editorial office of MDPI Antibodies, namely Arya Zou and Nathan Li, for their outstanding support.
